# Evaluating the Influence of the Microsatellite Marker Set on the Genetic Structure Inferred in *Pyrus communis* L.

**DOI:** 10.1371/journal.pone.0138417

**Published:** 2015-09-18

**Authors:** Jorge Urrestarazu, José B. Royo, Luis G. Santesteban, Carlos Miranda

**Affiliations:** Departamento de Producción Agraria, Universidad Pública de Navarra, Pamplona, Navarra, Spain; Wuhan Botanical Garden of Chinese Academy of Sciences, CHINA

## Abstract

Fingerprinting information can be used to elucidate in a robust manner the genetic structure of germplasm collections, allowing a more rational and fine assessment of genetic resources. Bayesian model-based approaches are nowadays majorly preferred to infer genetic structure, but it is still largely unresolved how marker sets should be built in order to obtain a robust inference. The objective was to evaluate, in *Pyrus* germplasm collections, the influence of the SSR marker set size on the genetic structure inferred, also evaluating the influence of the criterion used to select those markers. Inferences were performed considering an increasing number of SSR markers that ranged from just two up to 25, incorporated one at a time into the analysis. The influence of the number of SSR markers used was evaluated comparing the number of populations and the strength of the signal detected, and also the similarity of the genotype assignments to populations between analyses. In order to test if those results were influenced by the criterion used to select the SSRs, several choosing scenarios based on the discrimination power or the fixation index values of the SSRs were tested. Our results indicate that population structure could be inferred accurately once a certain SSR number threshold was reached, which depended on the underlying structure within the genotypes, but the method used to select the markers included on each set appeared not to be very relevant. The minimum number of SSRs required to provide robust structure inferences and adequate measurements of the differentiation, even when low differentiation levels exist within populations, was proved similar to that of the complete list of recommended markers for fingerprinting. When a SSR set size similar to the minimum marker sets recommended for fingerprinting it is used, only major divisions or moderate (*F*_*ST*_>0.05) differentiation of the germplasm are detected.

## Introduction

Plant genetic resources play a key role in sustainable agricultural production, so the need for preservation of endangered germplasm has encouraged collection programs and the formation of genebank collections worldwide. However, conserving plant genetic resources only fulfills its purpose when they are used effectively, which requires previous knowledge of the extent and structure of the variation occurring within the material preserved. An accurate fingerprinting allows detecting the redundancies that inevitably appear within and between collections [1–4]. Moreover, that information can be used to elucidate in a robust manner the genetic structure of germplasm collections, and then allow a more rational and fine assessment of genetic resources, focusing on a subset of accessions that could serve as representative of the entire genetic diversity available [5–8].

Microsatellite markers (SSRs) have been the most widely applied marker-type in the characterization of germplasm collections. In fact, lists of recommended SSRs have been proposed in the last years for several species in order to improve the management efficiency of the collections, and to allow cross-comparison between them [9–11], to solve questions about the identity of the germplasm under study [12,13] and to evaluate diversity [14,15]. Moreover, SSRs have also shown their robustness in the detection of the underlying genetic structure for a wide range of fruit tree species [4,5,16–20]. Therefore, despite SNP arrays for some of the most economically important fruit tree species are being released in the last years [21–24], and undoubtedly constitute a promising tool towards the identification of genomic regions associated with relevant horticultural traits and discovering new features for an efficient breeding, it is relevant to put in value the information generated in germplasm collections by SSR markers during the last years, the elucidation of the underlying genetic structure being a key point for that purpose [8].

The genetic structure of collections is nowadays majorly inferred using Bayesian model-based approaches [25–27]. Bayesian methods have overcome the traditional distance-based methods that, despite being relatively effective [8], suffer from several disadvantages [27]. On the one side, the clusters identified may be heavily dependent on both the distance measure and the graphical representation chosen and, on the other side, assessing the meaningfulness of the structure inferred and incorporating additional information is difficult. Among the wide set of Bayesian clustering methods available, Structure [27] is one of the most widely used as (i) it allows the user to easily adapt different analyses in a straightforward way with a unified approach [28]; (ii) it can handle codominant and dominant markers and allows the use of linked markers [29]; (iii) provides different ancestry and allele frequency models; (iv) allows performing inference of genetic structure in datasets that include several levels of ploidy; and (v) can assign individuals to populations without requiring previous information [30]. Structure analysis is therefore an effective method to analyze genetic materials such as fruit tree cultivars, whose assemblage, even when collected within a small area, cannot be strictly regarded as that of a biological population since it has been human-mediated.

One of the practical challenges for the study of genetic structure is that the number of markers required to infer it robustly is still largely unknown. This is particularly relevant since collection fingerprinting is frequently performed with a reduced set of optimized and robust markers that have shown to be highly efficient at that task, but whose ability for structure inference has not been proven. For instance, in genus such as *Malus* or *Pyrus*, as few as six SSRs can be enough to allow cross-comparisons between collections to detect duplicates and synonyms with little risk of misidentifying a genotype with a randomly chosen one taken from a larger sample [10,11]. For these genera, Bayesian analyses of genetic structure have been performed using between 8 and 20 SSR markers in *Pyrus* [20,31–35] and *Malus* [4,14,36–44], but there is no information on how the number of markers considered for inference affects the genetic structure revealed. In fact, to our knowledge, research on this specific topic has been only performed on humans or animals [27,45–48] and, in plants, only the work by Neophytou [49] uses relatively a similar approach for a totally different purpose (elucidate the genetic assignment and study of hybridization in oak).

The main objective of this study was to evaluate the influence of the number of SSR markers on the genetic structure inferred in *Pyrus communis* L. germplasm, also evaluating the influence of the criterion used to select those markers on the robustness of the genetic structure inferences.

## Materials and Methods

### Plant material and SSR genotyping

244 pear accessions were considered: 141 from the Universidad de Lleida (UdL) Germplasm collection described in Miranda et al [20], 61 accessions from the Public University of Navarra (UPNA) Germplasm collection, and 42 reference cultivars (Table 1). Reference cultivars were varieties bred in the 19^th^ century or earlier (mainly Northern European), or that included this kind of cultivars in their pedigree, and they were chosen to include widely diverse material in terms of origin and parentage. The full list of the material used, including accession names, sites of collection and collecting source codes according to Food and Agriculture Organization of the United Nations/International Plant Genetic Resources Institute (FAO/IPGRI, [50]) multicrop passport descriptors, is available in S1 Table.

**Table 1:** Pear cultivars used as reference in this study, indicating reported parentage, origin and group placement by Structure analysis.

Cultivar	Reported parentage	Origin^a^	Structure Group^b^
Cure	Unknown	France, 1760	**G1D**
Roma	Unknown	Italy, unknown	**G1D**
Abbé Fétel	Unknown	France, 1866	**G2D**
Beurré Alexander Lucas	Unknown	France, 1871	**G2D**
Beurré Bosc	Unknown	Belgium, 1807	**G2D**
Beurré d'Anjou	Unknown	France, 19th century	**G2D**
Beurré Giffard	Unknown	France, 1825	G2D
Bonne Louise d´Avranches	Unknown	France, 1870	**G2D**
Cascade	Max Red Bartlett x Doyenné du Comice	USA, 1986	G2D
Charles Ernest	Unknown	France, 1879	**G2D**
Concorde	Conference x Doyenné du Comice	UK, 1995	**G2D**
Conference	Seedling of Léon Leclerc de Laval	UK, 1894	**G2D**
Devoe	Seedling of Clapp's Favourite	USA, 1947	**G2D**
Doyenné du Comice	Unknown	France, 1849	**G2D**
Dr. Jules Guyot	Unknown	France, 1870	**G2D**
Epine Du Mas	Unknown	France, unknown	G2D
Général Leclerc	Seedling of Doyenné du Comice	France, 1950	**G2D**
Grand Champion	Russet Gorham sport	USA, 1943	**G2D**
Jeanne d'Arc	Beurré Diel x Doyenné du Comice	France, 1893	**G2D**
Maxine	Unknown	USA, 1845	**G2D**
Monsallard	Unknown	France, 19th century	**G2D**
Noveau Poiteau	Unknown	France, 1827	G2D
Packham´s Triumph	Uvedale´s St. Germain x Williams Bon Chrétien	Australia, 1897	**G2D**
Passe Crassane	Unknown	France, 1855	**G2D**
Pierre Corneille	Beurré Diel x Doyenné du Comice	France, 1849	**G2D**
Précoce de Trévoux	Unknown	France, 1862	**G2D**
Precoce di Fiorano	Beurré Giffard x Coscia	Italy	**G2D**
Président Drouard	Seedling of Beurré Napoléon	France, 1886	**G2D**
Rocha	Unknown	Portugal, 19th century	**G2D**
Super Comice Delbard	Unknown	France, 20th century	**G2D**
Tosca	Coscia x Williams Bon Chrétien	Italy, 1993	**G2D**
Triomphe de Vienne	Unknown	France, 1864	**G2D**
Wilder	Unknown	USA, 1870	G2D
Williams Bon Chrétien	Unknown	UK, 18th century	**G2D**
Winter Nellis	Unknown	Belgium, 1804	G2D
Abugo	Unknown	Spain, 20th century	**G4D**
Beurré Hardy	Unknown	France, 1820	G4D
Blanquilla	Unknown	Spain, 1747	G4D
Castell	Unknown	Spain, 19th century	**G4D**
Etrusca	Coscia x Gentile	Italy, 1992	**G4D**
Magallón	Unknown	Spain, 20th century	**G4D**
Flor de Invierno	Unknown	Spain	G4D

^a^ Reported parentage and origin of the most of the reference cultivars used in this study were consulted in the *Pyrus* Genetic Resources webpage of the USDA-ARS National Clonal Germplasm Repository (Corvallis, Oregon).

^b^Cultivars assigned with *Q*>0.8 indicated in bold.

Newly expanded leaves of each accession were ground to a fine powder in a microdismembrator (B. Braun Biotech International, Melsungen, Germany). Genomic DNA was isolated from 50 mg of this fine powder with Qiagen Dneasy Plant Mini kit (Qiagen, Hilden, Germany) according to the manufacturer´s instructions. DNA concentration of each sample was determined using a NanoDrop 2000 (Thermo Fischer Scientific, Wilmington, DE, USA), and DNA working dilutions of each sample were adjusted to 5 ng μl^-1^.

A set of 29 SSRs was used in this study (Table 2). Seventeen correspond to those included in the list proposed by the European Cooperative Program for Plant Genetic Resources (ECPGR) for the screening of accessions belonging to *Pyrus* genus, whereas the remaining twelve were chosen as they have been successfully used before in other pear diversity studies. The markers selected cover all pear linkage groups to ensure independence among loci. All of them were amplified in five multiplex polymerase chain reactions (PCR), denoted as A, B, C, D and E (Table 2).

**Table 2:** Microsatellite code, linkage group, PCR details and size range (bp) of 29 SSR loci analyzed in this study.

Locus^a^	Linkage group n°	Multiplex	Dye	Size range (bp)	Reference
CH-Vf1	1	B	VIC	129–172	Vinatzer et al. [51]
RLG1-1	1	E	NED	—	Yamamoto et al. [52]
CH02b10	2	B	PET	120–157	Gianfranceschi et al. [53]
CH03g07	3	A	VIC	203–267	Liebhard et al. [54]
NB109a	3	D	6-FAM	122–201	Yamamoto et al. [52]
NH023a	3	D	VIC	115–175	Yamamoto et al. [52]
NZ05g08	4	B	6-FAM	100–122	Guilford et al. [55]
CH01d03	4	B	6-FAM	129–185	Liebhard et al. [54]
CH04e03	5	C	6-FAM	176–206	Liebhard et al. [54]
NB103a	5	E	VIC	77–152	Yamamoto et al. [51]
CH03d12	6	B	NED	90–161	Liebhard et al. [54]
EMPc117	7	C	6-FAM	88–140	Fernández-Fernández et al. [56]
CH01h10	8	E	6-FAM	90–124	Liebhard et al. [54]
CH05a02	8, 15	A	NED	105–129	Liebhard et al. [54]
NB106a	9	D	NED	79–131	Yamamoto et al. [52]
NH029a	9	D	PET	85–104	Yamamoto et al. [52]
GD142	9	A	6-FAM	139–184	Hokanson et al. [57]
CH01f07	10	C	NED	172–219	Liebhard et al. [54]
CH02c11	10	B	PET	216–248	Liebhard et al. [54]
NB105a	11	D	PET	139–189	Yamamoto et al. [52]
EMPc11	11	C	VIC	138–160	Fernández-Fernández et al. [56]
CH01d09	12	A	PET	122–176	Liebhard et al. [54]
GD147	13	C	PET	125–162	Hokanson et al. [57]
CH04c07	14	A	VIC	—	Liebhard et al. [54]
CH01d08	15	A	PET	245–306	Liebhard et al. [54]
CH02d11	15	C	NED	97–145	Gianfranceschi et al. [53]
CH02c09	15	B	VIC	228–283	Liebhard et al. [54]
CH05c06	16	A	6-FAM	88–120	Liebhard et al. [54]
GD96	17	A	NED	—	Hokanson et al. [57]

^a^Underline indicates SSR markers recommended by the ECPGR [10].

PCRs for the A, B and C multiplex PCRs were performed in a final volume of 10 μl using 10 ng of DNA template, 1X PCR Master mix of QIAGEN kit multiplex PCR (Qiagen, Hilden, Germany) and 0.20 μM of each primer, except for CH02b10 and NZ05g08, for which 0.60 and 0.80 μM were used respectively, and for CH04c07 and CH03g07, for which 0.40 μM were used. The temperature profile for the three multiplexes was the one proposed by Evans et al. [10], but using an initial denaturation step at 95°C for 15 min and a final extension step at 72°C for 30 min. The reaction mixtures for D multiplex PCR were performed as is indicated above, but using 0.10 μM for all the primers and the following temperature profile: 95°C for 15 min, 5 × [95°C for 30 s, 57–52°C (−1°C/cycle) for 1 min, 72°C for 1 min], 30 × (95°C for 30 s, 52°C for 1 min, 72°C for 1 min), and a final step of 30 min at 72°C. The temperature profile for the PCR reactions of the three SSRs that composed E multiplex PCR, was conducted with an initial denaturation step at 95°C for 15 min, followed by 30 cycles of 30 s at 95°C, 1 min at annealing temperature and 1 min at 72°C, and a final 30 min extension step at 72°C. The annealing temperature used was 58°C for CH01h10, 42°C for NB103a, and 47°C for RLG1-1. PCR reactions were carried out in a thermal cycler (model 2720; Applied Biosystems, Foster City, CA, USA) and the fluorescently-labelled PCR products were separated by capillary electrophoresis using an ABI PRISM 3730 (Applied Biosystems, Foster City, CA, USA). PCR products were analyzed and sized with Peak Scanner Software ver. 1.0 (Applied Biosystems, Foster City, CA, USA).

### Influence of the number of SSR markers on the genetic structure inferred

Structure analyses were performed considering a variable number of SSR markers that ranged from just two up to 25, in order to evaluate how increasing the number of markers affected the genetic structure inferred. The order used to incorporate the SSR markers was not random, but based on the discrimination power showed by each marker and on the linkage group the marker belonged to. Thus, the markers showing the highest discrimination power (DP) were included first. However, markers belonging to a linkage group already included, regardless of its DP, were not added to the analysis until all the remaining linkage groups were represented. DP was calculated as defined by Tessier et al [58]:

$$DP=1-\sum_{i=1}^{I}p_{i}^{2}$$

where *p*_i_ represents the frequency of the *i* ^*th*^ banding pattern and *I* all the banding patterns generated by a SSR marker.

The mathematical procedure used for the inference of the genetic structure of the material was the model-based Bayesian clustering method implemented in Structure v2.2.3 [27]. In this study, diploid and triploid material was present, so Structure software was run using the recessive allele approach [59], encoding the individuals according to their ploidy as described in Stöck et al. [60]. We used a 7.5·10^4^ burn-in period and 2·10^5^ iterations for data collection, as these parameters resulted in high stability of the results with 10 runs per *K* value. The analysis was run for *K* values ranging from 2 to 10 inferred clusters and, in order to assess the best *K* value supported by the data, the *ΔK* method described by Evanno et al. [46] was used through Structure harvester ver. 0.6.93 application [61] to examine the rate of change in successive posterior probabilities over the range of *K* values. Additionally, the height of *ΔK* for the best *K* value supported by the data was used as an indicator of the strength of the signal detected by Structure [46]. When the results suggested that the *K* groups could be further structured in sub-groups (noted *K*_*S*_ for the sake of clarity), a second Structure analysis was performed individually for each *K* group [4,62–64], with 2 to 10 *K*_*S*_ inferred clusters explored. In such cases, to ensure that the variations on the inferred structure depended solely on the SSRs used, each sub-group was composed by all the genotypes except for all those with a membership value to another sub-group *Q*≥0.8. Therefore, some genotypes were analyzed for more than one sub-group. The final structure of the pear material was inferred with a subsequent Structure analysis using the population information obtained previously for the genotypes with a membership *Q*≥0.8 (PopFlag = 1) whereas no information (PopFlag = 0) was applied to those ones with *Q*<0.8. The placement of genotypes on groups or sub-groups was determined using CLUMPP ver. 1.1 [65], which evaluates the similarity of outcomes between population structure runs. CLUMPP output was used directly as input for Distruct ver. 1.1 [66] in order to generate barplots displaying the results.

Once these analyses had been performed, in order to determine the influence of the number of markers on the structure inferred, we computed for each number of markers the average of the highest membership coefficient of genotypes to a group or sub-group (*Q*). Besides, the stability (*D*_*i*_) of genotype assignments between marker numbers for a given *K* value as defined by Bouchet et al. [67] was also calculated:

$$D_{i}=\sqrt{\frac{\sum_{k=1}^{K}{\left(q_{ik}-q_{ik}^{\prime }\right)}^{2}}{K}}$$

where *q*_*ik*_ and *q’*_*ik*_ represent the assignment proportion of the genotype *i* to group *k* according to two different Structure analysis. This index was then used to calculate the average similarity index (*D*) between Structure analyses as:

$$D=1-\frac{1}{n}\sum_{i=1}^{n}D_{i}$$

where *n* is the number of genotypes.

Last, in order to compare group and sub-group differentiation as estimated with the increasing number of markers considered, *F* statistics were calculated including the genotypes assigned to different groups with an affinity *Q*≥0.8. Considering that the pear accessions in our study were diploid and triploid, the software Genodive v2.0b23 [68] was used to compute pairwise *F*_*ST*_ analyses with 10^3^ permutations to test for significance, as this software supports analyses of datasets containing individuals with different ploidy levels.

### Influence of the criterion used to select SSRs on the robustness of the genetic structure inferred

In order to test if the results obtained in the previous sections were influenced by the criterion used to order the SSRs, a validation study was performed. Two sorting criteria were compared: i) DP, in which SSRs were sorted according to their discrimination power as described previously and ii) *F*_*ST*_, in which sorting was made according to the value of the fixation index *F*_*st*_ for each SSR between the inferred populations. Once the SSRs were ranked according to their DP or *F*_*st*_, two choosing scenarios were considered: i) Most discriminant markers (or “best choices”), in which the selected ones had the highest values for each sorting criterion and, ii) Least discriminant markers (or “worst choices”), where the SSR with smallest values for the sorting criteria were selected. As in the previous analyses, rankings were also based on the linkage group the marker belonged; so that a linkage group was not repeated until all the remaining linkage groups were included. To ease calculations, the four possible combinations of sorting and choosing criterions were tested for two marker set sizes, 6 SSRs and 12 SSRs. The above-mentioned Structure procedure was applied on the validation datasets, and once the final structures of the pear material had been inferred, we computed the stability (*D*_*i*_) of genotype assignments between each validation data set and the full analysis with 25 SSRs. In order to compare the effect of the criteria used in the group differentiation, *F* statistics were also calculated, including the genotypes strongly assigned to the different groups (*Q*≥0.8).

## Results

### SSR polymorphism

The 29 SSR markers amplified in this study were polymorphic. Due to the poor amplification product, insufficient fluorescence signal or unreliable microsatellite profiles obtained when CH04c07, GD96 and RLG1-1 were used, we decided to exclude them of the study. Two out of the remaining SSRs, CH02c11 and CH05a02, amplified two loci located in two linkage groups as reported in Pierantoni et al. [69] and Garkava-Gustavsson et al. [70]. For CH02c11, the secondary locus was monomorphic, so only amplification for the main locus of this SSR was considered. The amplification range of CH05a02 in this study was from 105 bp to 129 bp, we decided to not consider it since it was very difficult to delimit the allelic range for each locus, so the study was finally performed using 25 markers distributed across 15 linkage groups (Table 3).

**Table 3:** Characteristics of the SSR markers sorted by their inclusion order in the overall Structure analyses.

				Alleles		Number of banding patterns	
Order of inclusion	Loci	Linkage Group	DP	Total	Effective	per marker	combined with previous markers
1	CH01d09	12	0.973	19	9.8	78	78
2	CH-Vf1	1	0.968	14	7.3	51	126
3	CH02b10	2	0.966	22	7.6	62	135
4	CH01f07	10	0.965	23	7.9	64	137
5	NB109	3	0.965	26	7.2	69	140
6	CH01d03	4	0.963	17	7.1	56	142
7	GD142	9	0.961	22	7.7	61	143
8	EMPc11	11	0.950	12	5.1	45	143
9	NB103	5	0.949	17	6.0	56	144
10	CH02d11	15	0.944	16	5.1	44	144
11	EMPc117	7	0.937	18	4.8	51	144
12	CH05c06	16	0.922	13	3.9	31	144
13	CH03d12	6	0.918	13	4.3	31	144
14	CH01h10	8	0.913	13	3.7	36	144
15	GD147	13	0.848	13	2.7	34	145
16	NB105	11	0.948	14	5.9	46	145
17	NH029	9	0.947	12	6.1	44	145
18	CH02c11	10	0.946	17	4.9	48	145
19	CH01d08	15	0.943	10	5.2	39	146
20	CH03g07	3	0.943	22	6.8	59	146
21	NZ05g08	4	0.942	10	5.4	41	149
22	NB106	9	0.877	17	4.0	47	151
23	NH023	3	0.876	18	2.9	27	153
24	CH02c09	15	0.874	12	2.5	33	155
25	CH04e03	5	0.566	9	1.7	12	155
*Mean*			*0*.*920*	*16*.*0*	*5*.*4*	*46*.*6*	
*Min*			*0*.*566*	*9*.*0*	*1*.*7*	*12*.*0*	
*Max*	* *	* *	*0.973*	*26.0*	*9.8*	*78.0*	* *

Discrimination Power (DP), alleles and banding patterns observed in the 202 pear accessions and 42 reference cultivars.

All the markers used, except for CH04e03 with a *DP* = 0.566, showed a high discriminant power, as the average *DP* = 0.920 and 20 marker had a *DP* above 0.9. The SSR markers are shown in Table 3 sorted by their DP values, in decreasing order. The average number of banding patterns per marker was 46.6, ranging from 12 (CH04e03) to 78 (CH01d09). As already shown by Tessier et al. [58], the order of markers according to the number of banding patterns they generated did not match the *DP* order (Table 3), given that the latter has into account not only the number of patterns, but also the frequency with which they appear. Using the 25 SSR markers, 155 genotypes were identified on the set of 244 accessions. However 5 SSRs sufficed to discriminate 90% of those genotypes (Table 3), whereas the 20 additional SSR markers allowed us to discriminate between the 15 last pairs of genotypes, which in most cases differed only in one allele, i.e. in less than 2% of the alleles analyzed per accession.

### Influence of the number of SSR markers on the genetic structure inferred

#### Number and robustness of populations detected

Results for the most probable *K* value detected in the Structure analyses depending on the number of SSR markers considered are detailed in S1 Fig, and summarized in Table 4. Irrespective of the number of markers considered, the best *K* value was *K* = 2 and, in most cases the signal indicating this value was very strong (*ΔK*>80). The results of Structure analyses using higher *K* values suggested extra sub-structuring of the diversity above that of *K* = 2, with individuals strongly assigned and asymmetric proportions found for each division level. Therefore, two subsample sets were formed and analyzed individually to further Structure analysis, each subsample set excluding from the whole set only those genotypes unambiguously assigned to the other (*Q≥*0.8). Using this criterion, we could include always the same genotypes on each subsample, ensuring that the variations on the inferred structure depended solely on the SSRs used.

**Table 4:** Influence of the number of SSRs on the number of groups and the robustness of the structure inferred.

	Complete Set		G1		G2	
n° of SSR	*K*	*ΔK*	*Ks*	*ΔK*	*Ks*	*ΔK*
2	2	16.6	6	3.0	2	0.8
3	2	135.3	3	31.9	3	8.3
4	2	169.9	4	31.3	4	6.0
5	2	82.0	3	12.6	8	3.0
6	2	48.7	4	76.3	3	6.9
7	2	167.3	5	29.6	3	12.6
8	2	122.5	4	223.0	3	6.5
9	2	164.2	3	39.8	3	6.2
10	2	106.2	4	49.9	5	12.4
11	2	168.5	3	23.3	2	39.1
12	2	232.5	3	42.1	2	43.3
13	2	116.5	3	41.9	2	46.6
14	2	168.4	3	66.5	3	7.5
15	2	235.1	3	54.3	5	5.4
16	2	238.1	3	53.2	4	3.1
17	2	130.3	3	121.2	2	43.4
18	2	141.8	3	97.9	2	50.3
19	2	84.8	3	160.2	3	7.7
20	2	189.7	3	54.4	4	21.1
21	2	133.2	2	73.6	3	6.0
22	2	35.1	3	153.4	3	6.9
23	2	2.4	3	70.8	3	9.5
24	2	85.3	3	68.0	3	12.2
25	2	82.9	3	72.7	3	10.6

Number of most probable groups (*K*), sub groups (*K*_*S*_) and strength of the structure signal (*ΔK*) detected within the complete set of genotypes and two major sub-structure groups (G1, G2) at increasing numbers of SSRs used for the inference.

Within the first group (G1), the highest likelihood for sub-grouping varied between *K*_*S*_ = 3 and *K*_*S*_ = 6 when the number of SSRs used was below 10, and stabilized at *K*_*S*_ = 3 for higher number of markers. Signal strength of the inferred structure was smaller than for the overall set of genotypes, but still high (*ΔK*_*S*_>30) in most cases, especially when more than 8 SSRs were used, and tended to increase with higher SSR numbers. Within the second group (G2), signal strength was generally much lower than in G1 (*ΔK*_*S*_<10 in 12 cases and only six with *ΔK*_*S*_>20). Moreover, the number of most plausible sub-groups within this group was generally modified when a new marker was introduced to the analysis, the most frequent *K*_*S*_ values were *K*_*S*_ = 3 (12 cases) and *K*_*S*_ = 2 (six cases), the latter showing the stronger signals observed within this subgroup. In accordance to these results, we explored *K*_*S*_ = 3 in G1 and *K*_*S*_ = 2 in G2 for the subsequent analyses.

**Fig 1:**
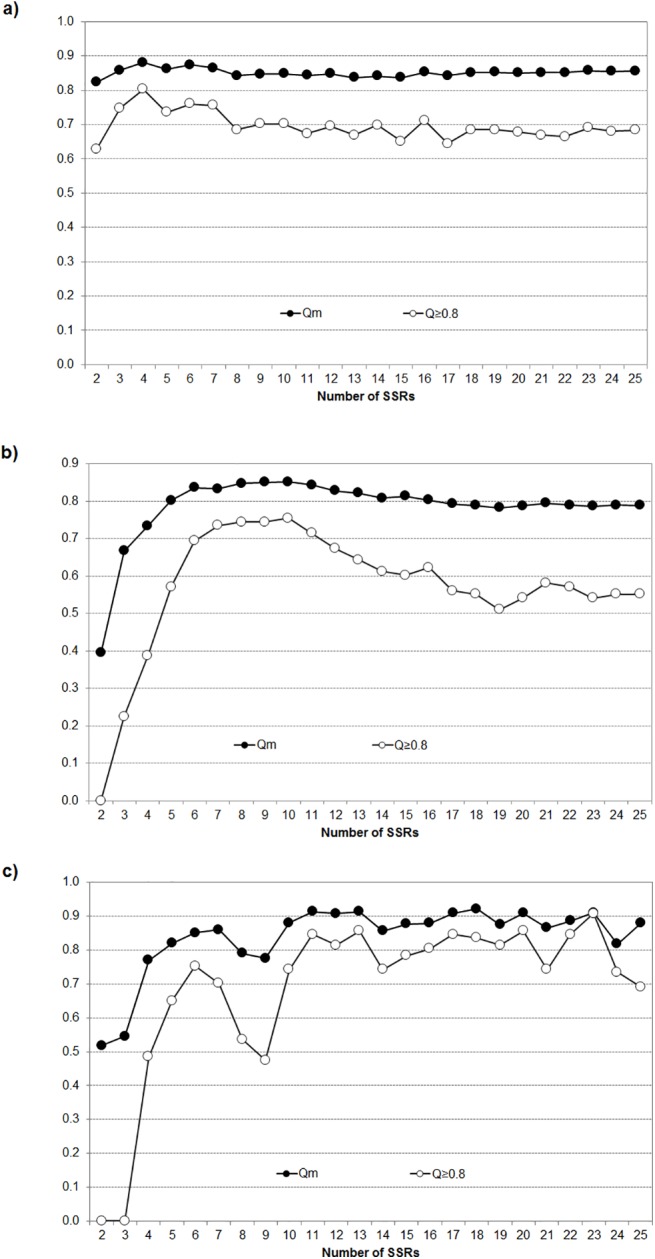
Influence of the number of SSRs on the assignation probabilities and the proportion of accessions strongly assigned. Exploration of mean assignation probability (*Qm*) and proportion of strongly assigned genotypes (*Q*≥0.8) at increasing numbers of SSRs used for structure inference. (a) Complete set of genotypes, *K* = 2. (b) major group G1, *K*_*S*_ = 3, (c) major group G2, *K*_*S*_ = 2.

To analyze the robustness of the groups and sub-groups obtained for the *K* and *K*_*S*_ values indicated above, simulations were examined to analyze the mean assignation probability (*Q*_*m*_) and the proportion of accessions assigned unambiguously to each partitioning level (Fig 1). The partitioning of the complete set of genotypes in *K* = 2 groups (Fig 1A) had always *Q*_*m*_ >0.8 and was nearly unaffected by the addition of SSRs to the analysis. However, the proportion of genotypes unambiguously assigned was maximum at 4 SSRs (80%) and stabilized around 67% from 8 SSRs onwards. For the three sub-groups in G1, *Q*_*m*_ increased steeply up to 6 SSRs (Fig 1B), and then stabilized around *Q*_*m*_ = 0.82. The proportion of strongly assigned genotypes showed a similar pattern up to 10 SSRs, and then decreased progressively. The general trends found for G2 (Fig 1C) were similar to those observed in G1, although more SSRs (11) were needed to reach the maximum values, and higher fluctuations were observed when incorporating a new SSR.

#### Stability of genotype assignment to groups

**Fig 2:**
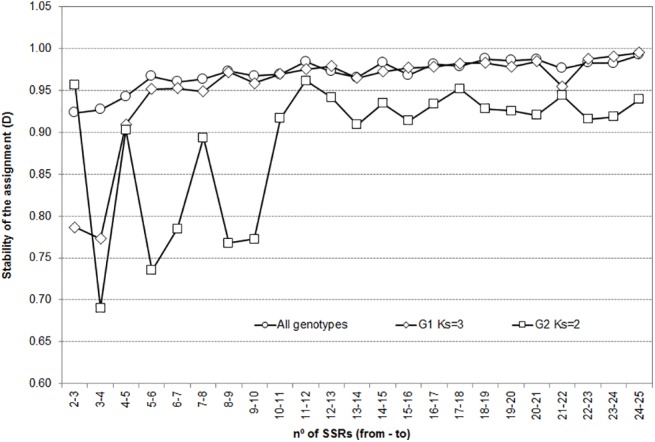
Stability of the assignment of genotypes to groups (*D*), increasing SSR number used in the inference one by one.

Results for the stability in the assignment depending on the number of SSR markers, when added to the analysis one by one are shown in Fig 2. For the complete set of genotypes, *D* was already very high (*D* = 0.92) when the third SSR was added, and increased progressively up to *D* = 0.995 for the 25^th^. For G1, the stability steeply increased as SSRs did, reaching *D* = 0.95 when the sixth one was added, and then the pattern was similar to the complete set. Assignments were very unstable for G2 when less than 10 SSRs were used; and for higher number of markers, *D* was consistently high (*D*>0.875) and fluctuations were less pronounced. The effect the number of SSR makers had on the stability of the assignments was analyzed adding 5 SSRs at each step forward (Table 5), it was also high (*D*>0.9) for the overall set and for G1, increasing as the starting number of markers did. A similar pattern was observed for G2, but at least 15 SSRs were needed to reach sufficient stability (*D*>0.9). Overall, *D* values below 0.9 implied that up to 30% of the genotypes were assigned to a different group when one additional SSR was included in the inference, and up to a quarter of these genotypes had been strongly assigned. For *D*>0.9 the change in probabilities when increasing the SSR set involved re-assignments for less than 8% of the genotypes and were seldom among the strongly assigned ones.

**Table 5:** Stability of the assignment of genotypes to groups (D), when the number of SSRs used in the structure inference is increased in more than one marker at a time.

	Structure population or sub-population		
n° of SSRs (from, to)	Complete Set	G1	G2
5–10	0.929	0.891	0.789
10–15	0.947	0.939	0.887
15–20	0.957	0.950	0.922
20–25	0.967	0.944	0.924

#### Estimation of population differentiation

**Fig 3:**
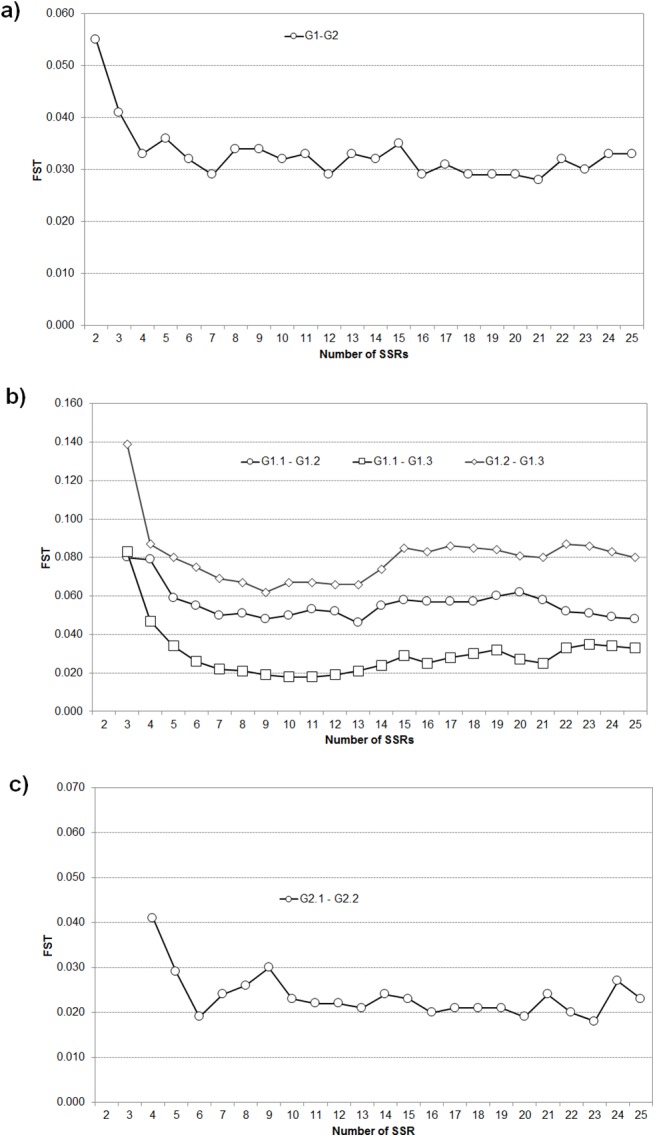
Influence of the number of SSRs used in the structure analysis on the pairwise differentiation values (*F*_*ST*_). **(**a) Complete set of genotypes, *K* = 2. (b) major group G1, *K*_*S*_ = 3, (c) major group G2, *K*_*S*_ = 2. Estimates of *F*_*ST*_ were always significantly different to zero in tests of 10^3^ permutations.

The influence of the number of markers used for structure analyses on the pairwise *F*_*ST*_ between inferred populations is shown in Fig 3. In all cases, *F*_*ST*_ estimates were significantly different to zero using 10^3^-permutation tests. Except for the whole set (Fig 3A), *F*_*ST*_ calculations could not be performed when two (in G1 and G2) or three (in G2) SSRs were used, as in those cases there were no genotypes with *Q≥*0.8. Generally, when less than 6 SSRs were used, the populations inferred appeared to be much more differentiated, with *F*_*ST*_ values up to three times those observed at higher SSR numbers. For more than 6 SSRs, in the complete set of genotypes a small differentiation (mean *F*_*ST*_ = 0.032) between the *K* = 2 groups was observed, with small variations in *F*_*ST*_ when a new SSR was added. Within G1 (Fig 3B), one group (G1.2) had a moderate differentiation with respect the others (mean *F*_ST G1.1-G1.2_ = 0.056 and *F*_ST G2.2-G2.3_ = 0.080), and another (G1.1) had little differentiation (mean F_ST G1.1-G1.3_ = 0.030). Additionally, *F*_*ST*_ for this set increased consistently for SSR>15. Within G2, partitioning in *K*_*S*_ = 2 groups (Fig 3C) showed little differentiation (*F*_*ST*_ around 0.020) and small variations when increasing SSR number, particularly for SSR<10.

#### Characteristics of the population groups inferred

**Fig 4:**
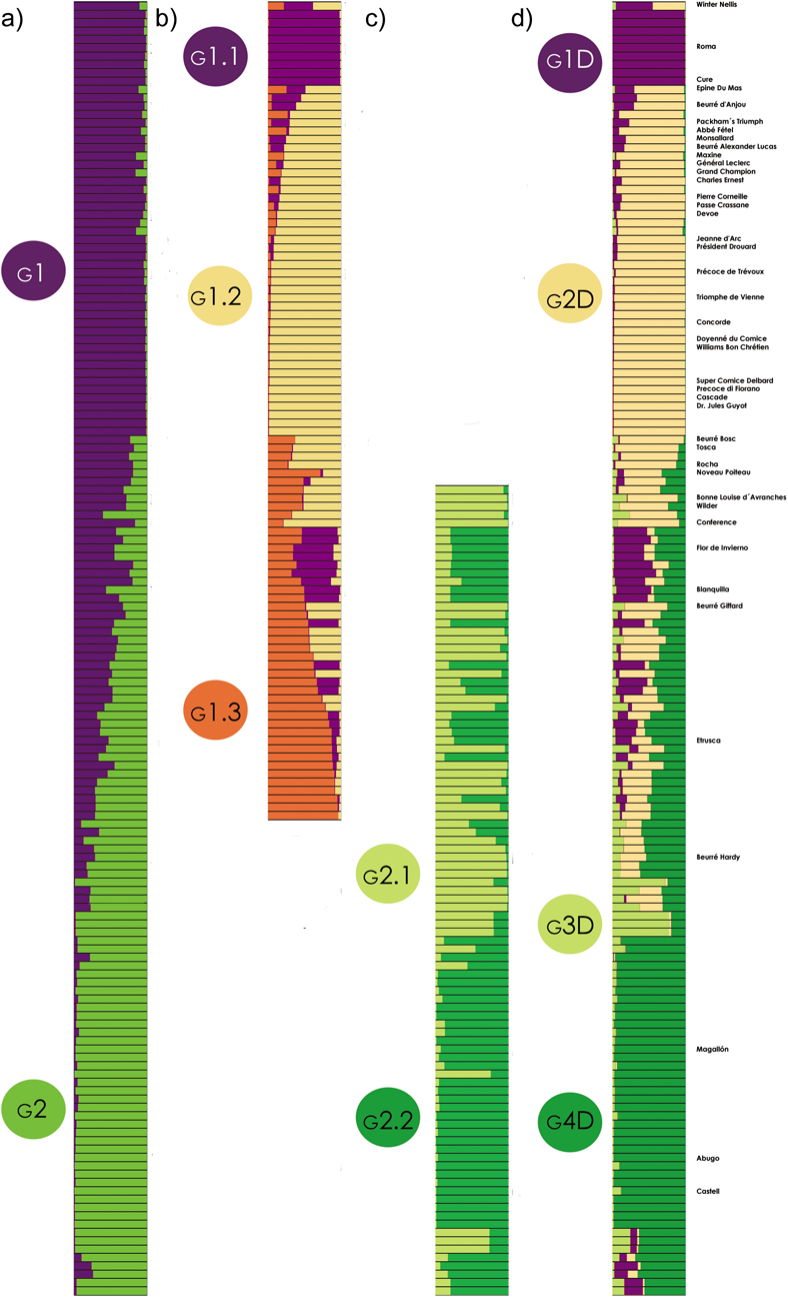
Substructuring of *K* = 2 Structure groups and placement of reference cultivars when 25 SSRs were used. (a) Structure analysis for the complete set of genotypes (b) nested Structure analysis for the first sub-group (G1), (c) nested Structure analysis for the second sub-group (G2) (d) Structure analysis for the complete set of genotypes using the prior information option, in which population information was added for the genotypes with membership *Q*≥0.8.

Structure barplots were generated for the partitionings inferred with 15 and 25 SSRs. Given that results were similar, Fig 4 shows only the results for 25 SSRs (a side by side comparison for both SSR numbers is provided in S2 Fig). The first level of partitioning (Fig 4A) clustered most (≈70%) of the collection genotypes in one group (G2) containing also all the Spanish reference cultivars (except ‘Flor de Invierno’) and the French ‘Beurré Hardy’, whereas G1 was composed equally of the rest of the reference cultivars and collection genotypes. Further partitioning of G1 (Fig 4B), revealed a group (G1.1) clustered around ‘Rome’ and ‘Cure’, another one (G1.2) containing most of the reference cultivars, whereas the third one was composed by the genotypes with *Q*_*G1*_<0.8 (in most cases, they were collection genotypes). The partitioning in *K*_*S*_ = 2 groups for G2 (Fig 4C) placed the references in different groups according to their origin, the Northern European ones were clustered in G2.1, and the Southern European in G2.2.

As the last step, the final structure of the collection was inferred with a subsequent Structure analysis using the *prior population information* option, in which genotypes were flagged with population information when they were strongly assigned at *Ks* = 3 partitioning levels of G1 and G2. In this case, the best results were obtained for *K* = 4 (Fig 4E). This analysis maintained nearly unaffected the clusters previously labeled as G1.1, G1.2 (now G1D and G2D, respectively), and merged G2.2 and some genotypes of G1.3 in G4D. The accessions in G2.1 that had been strongly assigned to G2 remained clustered in G3D, whereas the rest of genotypes, which were most of the loosely assigned (*Q*<0.65) in the initial Structure analysis, were shown to be in admixis. Mean differentiation among the four final groups was moderate (*F*_*ST*_ = 0.086), pairwise *F*_*ST*_ ranging from 0.069 to 0.144.

### Influence of the criterion used to select SSRs on the robustness of the genetic structure inferred

To ease calculations, the influence of sorting and choosing criteria on the robustness of the inferences were tested for the marker set sizes that had been identified as the minimum thresholds for a robust determination of the genetic structure reflecting major divisions in the germplasm (6 SSRs) and for a weaker structure with little, but significant, differentiation (12 SSRs). The markers used for each SSR choosing strategy are shown in S2 Table and the results for the most probable *K* values detected for each strategy are summarized in Table 6. Irrespective of the strategy, the best *K* value was *K* = 2, as in most cases the signal was very strong (*ΔK* >80). As previously observed, the results using higher *K* values suggested extra sub-structuring. For both groups, *K*_*s*_ values generally varied between *K*_*s*_ = 2 and *K*_*s*_ = 3, with moderate strength signals for G1 (20≤ *ΔK*_*S*_ ≤80) and somewhat lower strengths for G2 (10≤*ΔK*_*S*_ ≤60). When sorting criteria were compared, no clear trends appeared between DP and *F*_*ST*_, in some cases differences appeared for one of the parameters (number of groups detected, signal strength, assignation probabilities or proportion of genotypes strongly assigned), but they were not consistent between parameters, marker set sizes or groups. Overall, both sorting criteria offered quite similar results in their ability to detect and organize the genetic structure. Similar results could be observed when the choosing criterions were compared (Table 6), as the best and least suited markers offered similar results at both SSR marker set sizes.

**Table 6:** Influence of the SSR selection criterion on the robustness of the genetic structure inferences.

			Complete Set			G1			G2		
*SSR selection criterions*		n° SSRs	*K*	*ΔK*	*Q*_*m*_	*Ks*	*ΔK*	Q_m_	*Ks*	*ΔK*	*Q*_*m*_
Most discriminant	DP	6	2	82.0	0.873	4	31.3	0.837	3	6.9	0.851
	FST	6	2	139.4	0.873	2	30.0	0.809	3	27.7	0.753
	DP	12	2	232.5	0.848	3	42.1	0.828	2	43.3	0.907
	FST	12	3	64.5	0.823	3	20.6	0.782	2	61.1	0.870
Least discriminant	DP	6	2	85.0	0.881	2	8.6	0.786	2	3.0	0.640
	FST	6	2	57.3	0.853	2	76.6	0.793	2	9.5	0.812
	DP	12	2	229.0	0.851	3	23.6	0.797	3	14.1	0.796
	FST	12	2	266.0	0.896	3	4.2	0.698	3	14.1	0.811

Number of most probable groups (*K*), sub-groups (*K*_*s*_), strength of the structure signal (*ΔK*) and mean assignation probability (*Q*_*m*_) detected within the complete set of genotypes and two major sub-structure groups (G1,G2) with several criterions for choosing SSRs for the inference at two SSR marker set sizes (n° SSRs).

The influence of sorting and choosing criteria on the stability of genotype assignments to groups respect to the entire marker set was also compared (Table 7). In all cases but one (6 least discriminant SSRs in *F*_*ST*_ criterion) the stability was rather high (*D*_*i*_>0.83) and, as expected, it was better for 12 SSR marker sets (average *D*_*i*_ = 0.877) than for 6 SSR ones (average *D*_*i*_ = 0.823). Overall, slightly lower stabilities and higher proportion of genotypes assigned to different groups were observed for *F*_*ST*_, but the genetic structure inferred was mostly unaffected, particularly when looking at the strongly assigned genotypes, as at least 95% (for 6 SSRs) or 97% (for 12 SSRs) of the genotypes remained strongly assigned to the same genetic group when the entire 25 SSR set was used. Similar results were obtained when the choosing criterion was evaluated, but the inferred structure was slightly more affected, as the proportion of strongly assigned genotypes assigned to the same group dropped to 88% for 6 SSRs and 90% for 12 SSRs when the least discriminant markers were used.

**Table 7:** Influence of the SSR selection criterion on the stability of genotype assignments to groups.

				Proportion of genotypes assigned to a different group when 25 SSR are used			
		Stability (Di)		All genotypes		Strongly assigned	
*SSR selection criterion*		*6 SSRs*	*12 SSRs*	*6 SSRs*	*12 SSRs*	*6 SSRs*	*12 SSRs*
Most discriminant	DP	0.849	0.907	27.1	11.6	5.2	0.0
	FST	0.838	0.877	26.5	18.7	6.1	3.3
Least discriminant	DP	0.836	0.877	22.6	18.7	12.2	8.5
	FST	0.768	0.847	43.9	31.0	8.8	5.8

Stability of the assignment of genotypes to groups (Di) inferred with several criterions of SSR choosing at two marker set sizes, compared to the full analysis with 25 SSRs. Structure analysis was performed using the prior information option, in which population information was added for the genotypes with membership *Q*≧0.8.

Finally, the effect of the sorting and choosing criteria on the differentiation between groups is evaluated in Table 8. The average differentiation between groups ranges between *F*_*st*_ = 0.074 and *F*_*st*_ = 0.109 and, as happened in the increasing SSR number evaluation, in all cases *F*_*st*_ values are smaller when a higher number of markers was used. Irrespective of the choosing or sorting criterion used, the relative differences between group differentiation values tend to be maintained, and also can be observed that *F*_*ST*_ criterion tends to offer slightly higher differentiation values between groups than DP criterion.

**Table 8:** Influence of the criterion used to select SSRs for the Structure analysis on the pairwise differentiation values (F_ST_) between the inferred populations.

		6 SSRs				12 SSRs			
		DP		FST		DP		FST	
Populations	Full set (25 SSRs)	Most	Least	Most	Least	Most	Least	Most	Least
G1-G2	0.144	0.123	0.074	0.099	0.107	0.128	0.074	0.137	0.103
G1-G3	0.116	0.083	0.089	0.144	0.075	0.081	0.079	0.104	0.093
G1-G4	0.069	0.061	0.065	0.088	0.068	0.070	0.042	0.063	0.044
G2-G3	0.079	0.086	0.080	0.154	0.107	0.069	0.094	0.102	0.083
G2-G4	0.068	0.096	0.077	0.075	0.109	0.073	0.093	0.103	0.068
G3-G4	0.044	0.072	0.094	0.092	0.062	0.049	0.073	0.083	0.052
Average	***0*.*087***	***0*.*087***	***0*.*080***	***0*.*109***	***0*.*088***	***0*.*078***	***0*.*076***	***0*.*099***	***0*.*074***

## Discussion

### Fingerprinting efficiency of markers

Reliable markers are essential to fingerprint the accessions preserved in germplasm collections and to establish genetic relationships among them, helping the efficient management and use of the collections. As expected, each SSR used in this study (except for CH04e03), when considered alone, displayed a high degree of polymorphism and discriminant power. However, we must also consider the efficiency of the markers in combination with others, as it does not depend on discrimination power alone, but also on its independence from the set of primers already selected. For that reason, the lists of recommended markers aiming to standardize identification protocols include highly polymorphic markers placed in different linkage groups. In *Pyrus* and *Malus*, ECPGR recommends one per chromosome [10,11]. In these highly heterozygous species, it is possible to use even smaller marker sets to fingerprint germplasm collections at a global scale, while being reasonably sure that two accessions sharing a profile are at least closely related. For that reason, recommended lists include priority groups as it is acknowledged that not all laboratories would have sufficient funding to allow running the complete list. In our case, the five most discriminant SSR detected 90% of the genotypes identified with the complete set, but the remaining genotypes differed only in one of the alleles identified for the rest of the markers. We did not test the efficiency of all possible marker combinations, but the five least discriminant markers allowed us identifying nearly the same proportion (89%) of the genotypes. Overall, the markers used in this study display high efficiency for identification purposes, and as little as five would be enough to distinguish most of the genotypes of the set.

When more than 15 SSRs were used, we started to repeat linkage groups, so that four chromosomes (4, 5, 10 and 11) were represented twice, whereas three more (3, 9 and 15) were represented three times. This could raise some concern about the effect that the over-representation of some chromosomes could have on the inferred structure, as marker independence is a basic requirement in these studies [27]. However, it does not seem to be the case here, as we did not find noticeable differences among the results immediately below 15 SSRs and above that level. In fact, in all the cases where the placement in the reference linkage maps could be known [71,72], the distance between two loci was at least 20% of the linkage group length. Therefore, the results would be supporting all the markers in the set to be sufficiently independent.

### Influence of markers chosen on the structure inferred

Our main goal was to test the influence of the number of SSRs used on the ability of Structure to infer populations on a real *P*. *communis* dataset, with a secondary goal of testing the influence on that ability of the criterion used to select those markers. This kind of studies have been performed so far on human and animal populations (real or simulated), where a true structure based on the geographical origin of the genotypes can be assumed and, therefore, the inferences can be tested for accuracy comparing them with the real structure [46,47]. However, this approach is difficulted in most fruit tree species, as they are long-lived, and their distribution is widely human-mediated since the grafting has been the main traditional way to spread them since ancient times. The mode of reproduction and the human-mediated evolutionary processes have played a critical role in the genetic variation that it is possible to find nowadays in most of the fruit tree species. In a realistic scenario, a spatially and temporally dynamic process occurred over the course of the time while cuttings (and seeds) were exchanged between geographically distinct regions. Once in cultivated settings, the new materials contributed to diversify the existing *genepool* at each specific area, through inadvertent gene flow with other local cultivated individuals, or through directed breeding efforts characteristic of modern agriculture [73,74]. Thus, in species primarily propagated by clonal methods, geographical sampling information is less informative about their genetic structure, and cannot be considered to be *a priori* a reliable criterion to structure populations. For that reason, we have evaluated the ability of a marker set to provide reliable information about the genetic structure of *Pyrus* germplasm with a different approach: if the SSR set used is informative enough to give a reliable structure, the addition to the set of a new marker, independent to the already used, should not affect much to the structure inferred. Besides, the method used to select the markers appears not to be very relevant for structure inference.

The material used allowed us testing the influence of SSR number on three different scenarios: the first corresponded to the complete set of genotypes, which showed a very robust structuring reflecting major divisions in the germplasm, and the others appeared when searching for internal structure within the major groups. One (G1) had a strong sub-structure with moderate differentiation (*F*_*ST*_≈0.07), and the other (G2) a weaker sub-structure with little (but significant) differentiation (*F*_*ST*_≈0.03). Inferring population structure when differentiation between genetic groups is weak is relevant, especially on long-lived tree species that frequently exhibit high levels of within population variation but often weak population structure [73]. The latter value (*F*_*ST*_≈0.03) represents the minimum level of differentiation at which Structure has been reported to correctly infer genetic structure and assign individuals to their populations [75]. The strategy of allocating on each major group all the genotypes except for those strongly assigned to the other one has allowed us to obtain the different scenarios because the genotypes strongly assigned to any of the major groups were always the same. Therefore, the major groups were always composed by the same genotypes, and any change in the inferred structure depended only on the SSR set used.

The ability of Structure to detect a strong structure signal within the genotypes has depended on the differentiation scenario tested. Thus, the strongest signals (*ΔK*>150) were detected in the major division scenario, whereas the weakest (*ΔKs*≈10) were found in the little differentiation scenario. Both *ΔK* and *F*_*ST*_ values are seldom reported jointly in *Pyrus/Malus* genetic structure studies, but the few data available support this result: Iketani et al. [31] found *ΔK*>30 for Asian pear populations with *F*_*ST*_ up to 0.182, Urrestarazu et al. [4] in apple found major division (*F*_*ST*_ = 0.076) between germplasm with *ΔK*>1,200 and *ΔK* = 30 and *ΔK* = 100 for *F*_*ST*_ = 0.045 and *F*_*ST*_ = 0.115 using a nested model-based clustering approach, respectively, whereas Garkava-Gustavsson et al. [14] found *ΔK* = 4.02 for *F*_*ST*_ = 0.042. However, we did not detect any relevant influence of the number of SSRs used or the method used to choose them on the strength of the signal provided by Structure. In their simulations with human data, Evanno et al. [46] found an increase in signal strength when SSR set was increased from 5 to 10 markers, but in our case (Tables 4 and 6), once there were enough SSRs to detect structure, it always appeared although strong variations on the signal intensity could be observed.

Our results indicate that population structure and group membership could be inferred accurately once a certain SSR number threshold was reached, which depended on the underlying structure within the genotypes. In the scenario of a major division within the germplasm, as little as 2 SSRs were enough to find a strong structure signal, but four were needed to obtain also stable values of mean membership and differentiation. In the strong structure and moderate differentiation scenario, the combination of strong signal and stable allocation and differentiation were found for a minimum of 8 SSRs, that is, a similar level to the lower values found in the literature for *Pyrus/Malus* structure inferences [14,20,43,76], and somewhat higher than the minimum SSR numbers recommended for fingerprinting purposes [10,11]. However, for the weak structure scenario, this could not be accurately inferred until at least 12 SSRs were used. Overall, this suggest that in *Pyrus* (and probably also in *Malus*) genetic structure studies would be able to detect only major divisions and at best moderate differentiation between groups in germplasm if the analyses were performed only with the ‘high priority’ recommended marker sets. However, when highly variable loci, as SSRs, are used, even low *F*_*ST*_ values might indicate a biologically significant level of population differentiation [77]. Therefore, if the analysis aspires to obtain a robust detection of less differentiated populations (*F*_*ST*_<0.04), it would be advisable to use at least a SSR set of around 12–15 markers, i.e., a size comparable to the complete list of recommended SSRs for fingerprinting.

### Genetic structure of the *Pyrus* germplasm

This study has confirmed the clear genetic distinctness between the old and local Spanish accessions curated in germplasm collections and most of the cultivars used as reference found by Miranda et al. [20]. That study was performed using 8 SSRs, the accessions of the UdL collection and a smaller set of reference cultivars, also used here. The introduction of new accessions and reference cultivars has allowed us to detect a new group (split from the ‘old Spanish genebank genotypes’ group), and also confirms the rough correspondence between the geographic origin of the materials and their group placement, as most of the Northern European reference cultivars were clustered together, whereas the other clusters included mostly the local and ancient Spanish accessions and Southern European cultivars used as reference. It is noteworthy that Northern European cultivars remained clustered together even when Structure partitioning was explored at higher *K* values. A similar division between Northern European cultivars and local accessions was found by Ferreira dos Santos et al. [33] for Western Spanish pear germplasm, and also by Gasi et al. [34] when analyzed germplasm from Bosnia and Herzegovina. Overall, those results highlight the relevance of autochthonous pear germplasm as a reservoir of genetic diversity, and suggest that several differentiated *genepools* could be delineated within the European pear germplasm.

## Conclusions

The number of SSRs used affects the quality of a population structure inference in *Pyrus communis* L. germplasm, whereas the method used to choose them has a very minor influence. Marker sets including a number of SSRs similar to the minimum marker list recommended for fingerprinting this species can effectively characterize accessions, but, when used to infer genetic structure, they seem to be effective only at detecting major divisions or moderate (*F*_*ST*_>0.050) differentiation of the germplasm. In order to ensure that Structure analyses provide strong structure signals, robust structure inferences and adequate measurements of the differentiation, even when low (but significant) differentiation levels exist within populations, it would be advisable to use at least a similar number of SSR markers to the included ones in the complete list of recommended markers for fingerprinting. Additionally, the results of this study allow confirming a clear differentiation of European pear germplasm based on broad geographical lines and suggest that several *genepools* could be delineated within the material evaluated, highlighting the interest of the elucidation of the genetic structure in *Pyrus* germplasm at European high-scale. An integration of the data from collections from different European geographic regions will make possible this, which might be an important starting step to define the European “pear core collection” that it could be further used for association studies to identify genomic regions associated with important horticultural traits in this species.

## Supporting Information

S1 Dataset SSR profiles for the 155 unique genotypes used in this study. (XLSX)

S1 Fig Exploration of *K* values for Structure analysis of pear germplasm. The exploration was made by estimates of the ratio of the slope of the likehood curve (*ΔK*) calculated according to Evanno et al. [45] plotted against *K*.
(TIF)

S2 Fig Substructuring of *K* = 2 Structure groups and placement of reference cultivars when 15 and 25 SSRs were used. (TIF)

S1 Table Pear accessions included in the study. Collection information includes accession name and collection code, site of collection, specific latitude and longitude, approximate elevation and collecting source code according to Food and Agriculture Organization of the United Nations/International Plant Genetic Resources Institute [50] multicrop passport descriptors.
(XLS)

S2 Table Markers included in the validation datasets. (XLS)
