# Supplementary figures and images for: Evaluating the Influence of the Microsatellite Marker Set on the Genetic Structure Inferred in Pyrus communis L
Source: PLoS One. 2015 Sep 18;10(9):e0138417. doi: 10.1371/journal.pone.0138417 (PMC4575082; doi:10.1371/journal.pone.0138417)

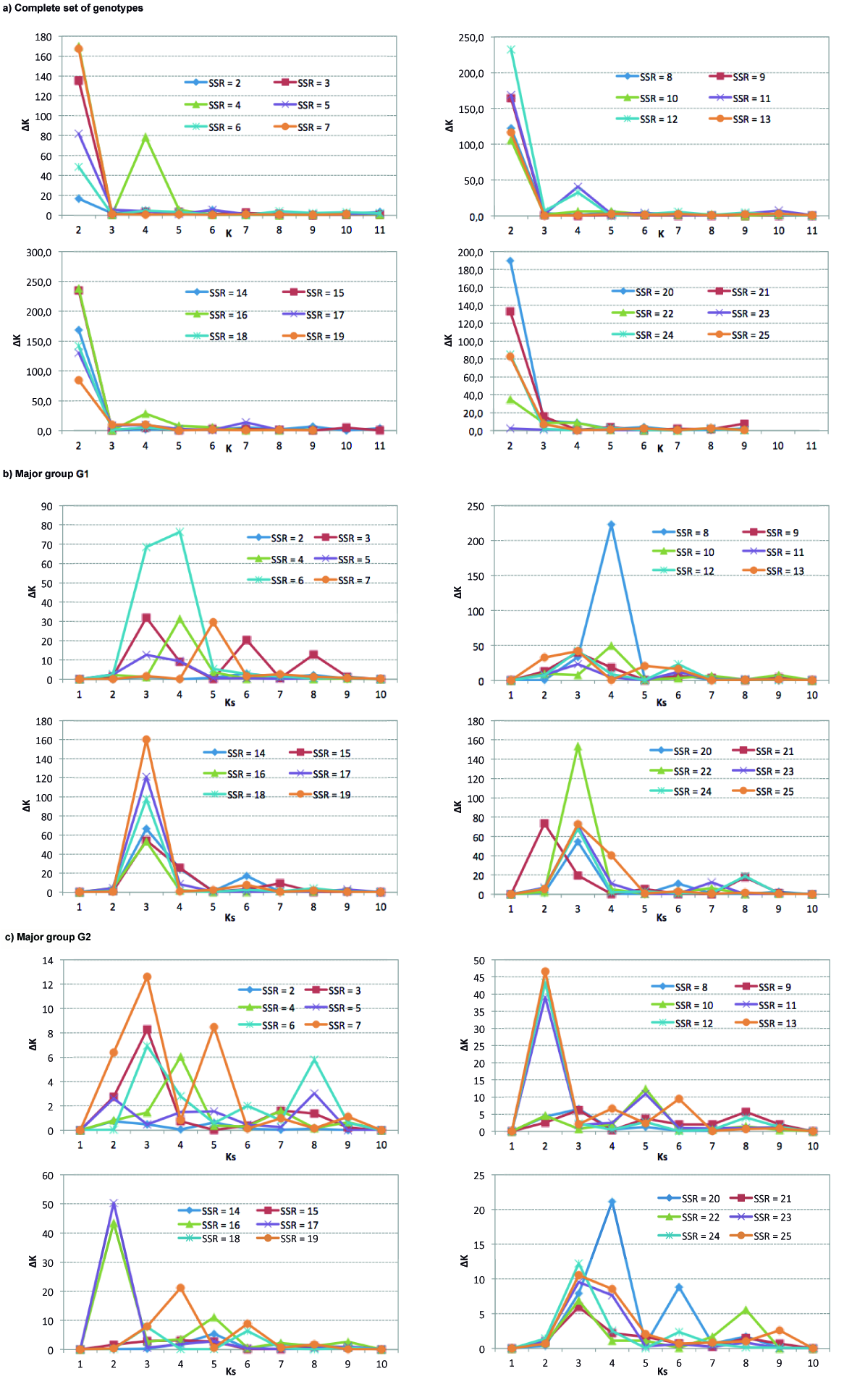

Supplement: S1 Fig — The exploration was made by estimates of the ratio of the slope of the likehood curve (ΔK) calculated according to Evanno et al. [45] plotted against K. (TIF) [file pone.0138417.s002.tif]

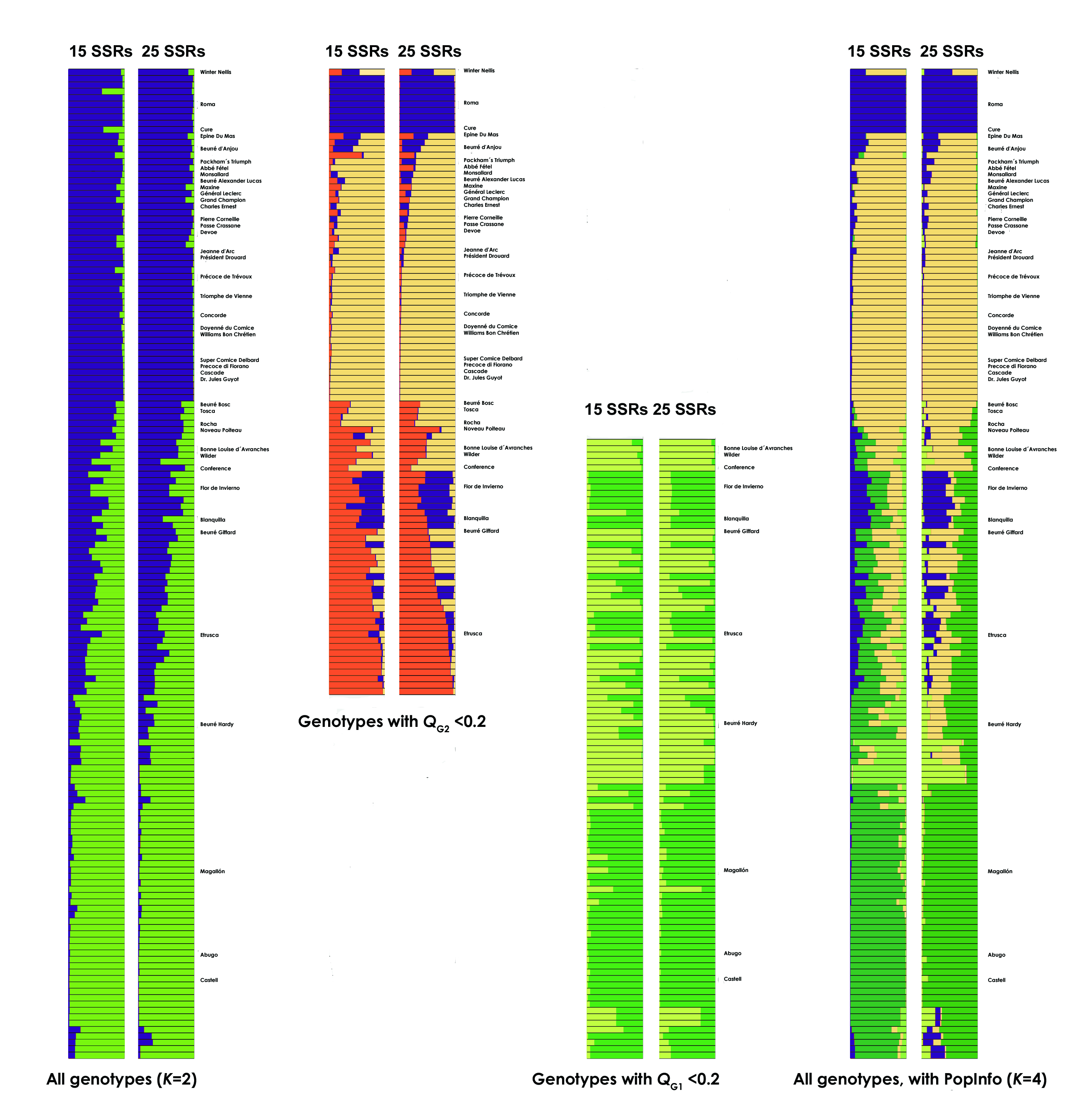

Supplement: S2 Fig — (TIF) [file pone.0138417.s003.tif]
